# Cross-Platform Comparative Study of Public Concern on Social Media during the COVID-19 Pandemic: An Empirical Study Based on Twitter and Weibo

**DOI:** 10.3390/ijerph18126487

**Published:** 2021-06-16

**Authors:** Wen Deng, Yi Yang

**Affiliations:** College of Public Administration, Huazhong University of Science and Technology, Wuhan 430074, China; dengwen@hust.edu.cn

**Keywords:** social media, COVID-19, Twitter, Weibo, crisis lifecycle, opinion leader

## Abstract

The COVID-19 pandemic has created a global health crisis that has affected economies and societies worldwide. During these times of uncertainty and crisis, people have turned to social media platforms as communication tools and primary information sources. Online discourse is conducted under the influence of many different factors, such as background, culture, politics, etc. However, parallel comparative research studies conducted in different countries to identify similarities and differences in online discourse are still scarce. In this study, we combine the crisis lifecycle and opinion leader concepts and use data mining and a set of predefined search terms (coronavirus and COVID-19) to investigate discourse on Twitter (101,271 tweets) and Sina Weibo (92,037 posts). Then, we use a topic modeling technique, Latent Dirichlet Allocation (LDA), to identify the most common issues posted by users and temporal analysis to research the issue’s trend. Social Network Analysis (SNA) allows us to discover the opinion leader on the two different platforms. Finally, we find that online discourse reflects the crisis lifecycle according to the stage of COVID-19 in China and the US. Regarding the status of the COVID-19 pandemic, users of Twitter tend to pay more attention to the economic situation while users of Weibo pay more attention to public health. The issues focused on in online discourse have a strong relationship with the development of the crisis in different countries. Additionally, on the Twitter platform many political actors act as opinion leaders, while on the Weibo platform official media and government accounts control the release of information.

## 1. Introduction

Social media platforms such as Twitter and Weibo are regarded as interactive communication tools for public debate and discourse [1] that reduce the cost of communication between users [2,3]; thus, individuals can express their own opinions on public issues and interact with likeminded people spontaneously [4]. The huge volume of data available on social media can help us understand the feelings of users about a certain issue or event, as these platforms store individual discourse. Additionally, online discourse about the same issues can be different on different social media platforms due to users’ differing societies, politics, and cultures. However, existing studies of social media have been conducted using different methods in different countries, while parallel comparative studies conducted using the same methods in different countries to identify similarities and differences are still rare [5]. It is particularly noteworthy that the Chinese state separates its citizens from the rest of the world through its firewall; this information control method may influence the expression of online discourse [6]. Due to this reason, although Chinese social media users occupy an important position in the global Internet, they have received relatively less attention [7]. Moreover, few issues generate worldwide interest, so the opportunities to perform comparative study in different countries are also limited.

Since the end of 2019, the outbreak of coronavirus (referred to as COVID-19) has caused a global public health crisis which has had a serious impact on international society. Unlike other types of emergencies, public health events do not affect a fixed population and are difficult to predict in terms of area and mode of occurrence [8]. The COVID-19 pandemic provides a suitable example for a comparative thematic research. As individuals around the world are stakeholders and risk takers under direct threat, they have paid attention to the development of the COVID-19 pandemic and taken part in intense online discussions. These online discourses contain references to personal emotions and valuable information from different countries [9], but little research attention has been given to the similarities and differences of online discourse in different countries during the COVID-19 pandemic.

This article applies text analysis techniques to analyze two social media platforms, Twitter and Weibo, and provides cross-platform comparisons of the discourse in these two social media ecosystems. We selected these two platforms as they are the most widely used social media platforms in both the English-speaking world (particularly in the US, where there are 48.35 million monthly active Twitter users, representing around 22 percent of US adults) and the Chinese-speaking world (in China, there are 511 million monthly active users of Sina Weibo—namely, Weibo—representing around one third of the population).

Although many scholars have conducted creative studies based on the COVID-19 pandemic or other representative public emergencies, most of them focus on the construction of emergency governance systems and departmental response strategies at the macro levels and do not pay enough attention to the reaction of people on the Internet [10,11]. In addition, there is a lack of research on data mining with regard to online public opinion and the visualization/measurement of large samples of data. Scholars tend to focus more on the problems of their own countries or certain regions, ignoring the diverse cultural backgrounds involved. There have been few empirical studies that have comparatively investigated the pandemic in different national contexts. It is difficult to clarify the elements of different countries that affect online public opinion or to propose strategies to identify opinion leaders.

Based on this, the core aim of this paper was to discover the similarities and differences in terms of online discussions and social networks between two platforms from two different countries. Is it possible to find an appropriate method to effectively conduct such a comparative study? In the face of the COVID-19 outbreak and continuously changing online environment, how can we measure social media users’ opinions and identify opinion leaders? Additionally, how can we scientifically explore the changing patterns of Internet topics involving the COVID-19 pandemic in the global context? Therefore, using the technique of data mining, this study takes Twitter and Weibo—two global social media platforms—as its research objects to compare the evolving trends of users’ opinions and hot topics in different periods, outline the patterns of information communication, and identify the leaders of online opinion. We explore the core nodes and development logic that affect the attention of users of the two platforms and respond to the concerns of online opinions about COVID-19.

Informed by social media’s function, characteristics, and opinion leaderships, our primary interests focus on several substantive research questions: What are the main issues that have been hotly debated on Twitter and Weibo during the COVID-19 pandemic? Are there any similarities and differences between Twitter and Weibo concerning the salient issues during different periods of the crisis? What are the characteristics of the social network structures of the different platforms? Who (account/user type) are the online opinion leaders on the two platforms?

## 2. Literature Review

### 2.1. Comparison between the Twitter and Weibo Platforms

Social media are used incrementally to promote citizens to express different opinions [12], which enables the public to play a more active role in the running of the country [13]. Social media platforms with great influence can morph from information networks into social networks [14]. This can be seen as the development of collective intelligence through an online collaborative approach to create content; it also evolves rapidly and plays a profound role in people’s perceptions and attitudes [15]. Among the different social media platforms, Twitter has proven to be a strong force in promoting international discourse due to its open, horizontal, and networked architecture and has already been extensively studied [16]. Weibo is regarded as the counterpart of Twitter in China. It has hundreds of millions of users and plays a foremost role in discourse in China, but has received relatively less attention from around the globe [17].

Some prior studies have compared Twitter and Weibo [17,18,19,20]. Twitter has been blocked in China since 2009. The users of the two platforms are mostly divided by China’s firewall, which separates the online social network users of China and those of the rest of the world (mainly users in the US in this research) [20]. The two platforms share many similarities in terms of influence, function, and structure, but barely overlap [17]. The two platforms are situated in two completely different political and social environments—liberal democracy versus state-regulated authoritarian. While we may assume that the different platform features may influence the nature of users’ behavior, few studies have actually compared the characteristics of social media in different cultures and countries [21].

Some comparative studies have investigated this issue from different perspectives. For instance, Han et al. [17], through an analysis of social networks, found that interaction between users of Weibo is weaker than that between users of Twitter. Bolsover and Howard [19] discussed the propaganda strategies and algorithms used on the two platforms and discover more evidence of automation on Twitter than on Weibo. Lin et al. [22] compared users’ information communication behaviors on Twitter and Weibo during extreme climatic events and found that Twitter users tended to use more humorous expressions than those of Weibo, but that the latter showed more organizational participation. Gao et al. [23] analyzed individuals’ habits and explored the cultural differences between the two platforms. They concluded that Twitter users (mainly from the US, which tends towards individualism) are more willing to present their posts for public discussion but that they are less positive than Weibo users (from China, which tends towards collectivism). Kim et al. [24] carried out a quantitative content analysis and suggested that US Twitter users tend to engage in mockery and political expression more than Chinese Weibo users, who rarely disagree with the government or its politics. However, there has been limited research focusing on an international event that affects individuals from different countries in an equal way. Thus, these comparative studies lack some control variables and little is known about how the dynamics of certain events may affect different online environments.

### 2.2. Online Discourse on COVID-19

When public crises occur, it is well known that individuals tend to seek immediate information through social media, especially in public health crises that have a lasting and widespread impact. Social media platforms have become the focus of many studies, as they have evolved into new legitimate sources of news and information and been increasingly relied upon by the public and officials when dealing with a crisis [22]. Except for official news, another main use of social media is seeking and spreading interpersonal information, as well as social support [25]. Social media provide a wealth of information on public emergencies on a micro level. On the one hand, social media can be used as a data source for detecting and tracking unexpected public events, such as the outbreak of a disease. Typical examples include Google Flu Trends, a real-time allergy surveillance system [26], and Twitter surveillance of the Ebola outbreak [27]. On the other hand, some studies have used social media to assist in public emergency management [28]. Thus, data mining from social media platforms is based on the growing awareness of its ability to provide insight into public opinion, extract hidden information, and identify trends. In public health crises such as epidemics, social media can be used to investigate public awareness, attitudes, and reactions [29].

Due to the outbreak of COVID-19, many individuals have been staying in isolation or total lockdown and social media have thus become their main means to share their experiences or concerns [30]. As such, a number of researchers have conducted studies based on the data from social media platforms [30,31,32,33,34,35,36]. For instance, Wicke and Bolognesi [31], through a topic analysis of users’ tweets, found that Twitter is generally used for “communications and reporting, community and social compassion, politics and reacting to the epidemic”. Meanwhile, social media platforms have also become prominent public spheres for debate. Some celebrities and public figures use social media to express their opinions. Thelwall and Thelwall compared the tweets of male and female Twitter users, suggesting that male users prefer to talk about sports cancellations, the global spread of the virus, and political reactions. Meanwhile, they found that female users pay more attention to family, social distancing, and healthcare [32]. Through text analysis, Goel and Sharma [30] identified some highly influential users during the debate around COVID-19 on Twitter and categorized these leaders into four clusters: research, news, health, and politics.

Meanwhile, some Chinese scholars have conducted a large amount of work on researching information flow on the Weibo platform. Cao and Yue [33] used topic mining and evolution analysis in their study and suggested that Weibo users keep a close eye on the development of the COVID-19 pandemic. They also stated that there were significant differences between the topics discussed in each stage of the COVID-19 pandemic. Li et al. [34] found that during the COVID-19 pandemic, Weibo users tended to show more concern more about personal health and their family members and focused less on leisure and friends. They also stated that the social media data suggest an increase in negative emotions and sensitivity to social risks in China. Li et al. [35], through analyzing the behaviors of Weibo users from Wuhan, discovered a positive correlation between user attention level and crisis severity level in Wuhan during the early stages of the COVID-19 pandemic and discussed the potential value of using social media data to predict real-world public health statistics. Su et al. [36] compared Weibo users in Wuhan and Twitter users in Lombardy in an attempt to explain public reactions and psychological states in the context of COVID-19. In summary, discourse content and user behavior analysis of social media can reveal the development trajectory of public attention during the COVID-19 crisis. However, most previous case studies have typically investigated a single linguistic or ethnic social media platform; cross-cultural comparisons of crisis communication using social media are scarce [22].

### 2.3. Theory Guiding: Crisis Lifecycle and Opinion Leaders

Scholars have found that online discourse bears a strong relation to news reports as well as the development of a crisis [37]. The public gradually regard social media as a means to make sense of news in times of crisis [22]. Considering that there was a time lag in the development of the COVID-19 pandemic between the US and China, in order to analyze the topics trend of online discourse according to the development of the COVID-19 crisis we introduced lifecycle theory as a guideline. Scholars have studied the lifecycle of a crisis from various perspectives; for instance, in 1986 Fink [38] provided a four-stage map of a crisis, which included the prodromal crisis stage, the acute crisis stage, the cornice crisis stage, and the crisis resolution stage. As not all crises can be divided into four stages, Pearson and Mitroff [39] suggested that a crisis goes through five stages: signal detection, preparation/prevention, containment/damage limitation, recovery, and learning. After a comparison and combination of Fink and Mitroff’s models, Coombs [40] suggested a three-stage model, which included the pre-crisis, crisis, and post-crisis stages. Some existing studies have used one or more crisis lifecycle models and discovered the law of development [41,42,43].

To better analyze the structure of a social network, we introduced the opinion leader concept. An opinion leader can be regarded as a person who can reach out to and inspire a large number of people; these people are important actors in the diffusion of information and can significantly affect individuals’ behavior [44]. Opinion leaders are individuals who are strategically located in the nexus of information flows and whose remarks are influential. Opinion leaders can generate many discussions to gain support from other actors [45]. They also apply social support and social pressure to influence their networks [46,47]. Their presence facilitates the process of opinion building at the periphery, and they can strategically transfer information to more passive and less central members of the network [48,49]. Unsurprisingly, these characteristics are replicated in digital environments (including social networks), but in these environments, traditional forms of social hierarchies and cues are replaced with other attributes that confer influence [50]. Opinion leaders with more knowledge resources might be better able to engage in producing content than those who are not opinion leaders [51,52,53].

Overall, the communicative content and structures of social media platforms such as Twitter and Weibo are determined by two overlapping and interdependent networks. However, in the extant literature only a few scholars have performed a cross-national comparative analysis; most studies instead compare different media within a single country to determine factors such as partisanship or compare media in different countries in order to frame the same event or group. Scholarly studies that examine both social media in different societies and various forms of media within a given society are very scarce [54,55].

## 3. Methods

### 3.1. Data Collection

In this study, we selected comments from users of Twitter and Weibo as our dataset. Firstly, we determined the time frame of the sample. Relevant tweets (posts) on Twitter and Weibo were collected between 9 February and 10 March 2020. This was a period when the number of daily new confirmed cases in both China and the US were at a high level (https://covid19.who.int/, accessed on 10 January 2021) and is representative enough to reflect the public reactions to COVID-19. In addition, several important and controversial international events took place during this period and people’s fears and discussions about the epidemic were reflected in their social network posts, which are significantly representative. We used *Houyi* (https://houyi-caiji.oss-cn-beijing.aliyuncs.com/update/zh-CN/nature/houyicaiji-setup-3.6.0.exe, accessed on 10 January 2021), a powerful web crawler tool equipped with an IP pool to guarantee the effectiveness of the data crawling. *Houyi* is also an intelligent tool that can capture comments and identify users’ information, such as their region or gender. We collected text from Twitter and Weibo—namely, American and Chinese users’ comments about COVID-19. Additionally, we decided to collect and analyze only tweets/posts that contained specific keywords following these three steps:

(1) Identify keywords on different platforms. Keywords on Twitter = “coronavirus”, “COVID-19”; keywords on Weibo = “新型冠状病毒肺炎”, “新冠肺炎”.
(2) Data in the two ‘ecosystems’ were collected at the same time each day: 11 a.m. China standard time and 10 p.m. EST in the US.
(3) The comments collected from users were collected based on four common items on the platform: users’ id, content, publishing time, and geolocation [56].

After an initial data cleaning to remove emojis and comments in other languages, the sample size included N = 101,271 tweets (more than 2 million words) and N = 92,037 Weibo posts (more than 26 million words). To better arrange the data, we divided the comments in one document into three different days, thus obtaining two corpora representing the Twitter and Weibo platforms, with each corpus containing 10 documents.

### 3.2. Data Preprocessing

Before the text analysis, we needed to complete some pre-processing operations, including word segmentation (mainly for Chinese text), data cleaning, and weight calculation. These operations were implemented using the open-source Python scripting language and combined with manual correction (https://blog.csdn.net/Eastmount/article/details/104698926#_161, accessed on 10 January 2021).

**Word segmentation.** When dealing with a Chinese corpus, the text may be incorrectly segmented due to the lack of reference vocabulary [57]. The most effective way to solve this problem is to use a more extensive dictionary to enhance the vocabulary collection. We introduced the most popular Chinese word segmentation module *Jieba* to segment each sequence; this module has been widely applied in text analysis.

**Data cleaning.** We expanded the basic English and Chinese stop-word lists based on our corpus features to accomplish stop-word removal. Due to certain limitations, *Jieba* could not fully identify some typical Chinese words. To complete the task of stop-word removal, we utilized a list of Chinese stop-words to conduct manual adjustment (https://gitee.com/UsingStuding/stopwords, accessed on 15 January 2021). Thereafter, each public comment document was represented as a vector of words. In addition, we performed a conversion of the language characters and eliminated punctuation.

**Weight calculation.** We used Term Frequency-Inverse Document Frequency (TF–IDF) to calculate the weights. This is the most fundamental form of document representation for transforming unstructured documents into a structured numerical vector and it supports language processing in both English and Chinese. TF-IDF is based on the Bag of Words scheme, in which a document can be represented by a collection of words used in the document. The method also assumes that if a word is important in a document, it should repeatedly appear in this document and not in other documents, which is why the TF-IDF produces statistics, term frequency (hereafter TF), and inverse document frequency (hereafter IDF). This step was implemented using the Python scripting language (https://blog.csdn.net/Eastmount/article/details/104698926#TFIDFKMeans_456, accessed on 15 January 2021).

The parameter tf*_ij_* is defined as the number of times word *i* appears in document *j*. The value is larger if the word is more important. The parameter df*_i_* is the number of documents in which word *i* appears at least once. The larger the value is, the more common the word is. If a word *i* is assumed to be important in document *j*, it should have a large TF (tf*_ij_*) and a small DF (df*_i_*). The formula of TF-IDF is as follows:

$$\mathrm{TF}-{\mathrm{IDF}}_{ij}={\mathrm{tf}}_{ij}\times \log\left(\frac{N}{{\mathrm{df}}_{i}+1}\right).$$

### 3.3. Structure Topic Model with LDA

The topical model is a methodology that is widely used in natural language processing, topic discovery in disordered documents, and semantic mining. The most common topic probability model used in social science is Latent Dirichlet Allocation (LDA) [58]. As the method is automatically implemented based on mathematical algorithms, it can minimize subjective deviation when analyzing data. LDA has been widely applied in politics, economy, and entertainment in different countries [59,60,61]. Inspired by these methods, this research used LDA to implement a text analysis to identify the topics discussed in the collected data.

The LDA process is based on text information in a corpus that is generated by the public in tweets or posts about diverse aspects of COVID-19, as stored in the website backend database. The topical modeling by LDA in this study used the following steps:

**LDA programming.** The LDA analysis processing in this paper was implemented by open-source Python programming (https://blog.csdn.net/Eastmount/article/details/104698926, accessed on 15 January 2021). This allowed us to find typical lexical units (LDA words) and topics (LDA topics) in a subset of the corpus by extracting the underlying context to identify the users’ discourse.

**The number of topics.** We selected the number of potential topics as K = 15, 20, 25, 30, 35. According to other studies, if the number of topics is too small, terms that originally belong to different topics will be grouped under the same topic; if the value is too large, terms that should belong to the same topic will be assigned to different topics. In this study, after a series of comparisons we found that the extraction efficiency was highest when the total number of classified topics for Twitter is K = 35 and K = 30 for Weibo.

**The number of iterations.** This number is the number of iterations needed to perform Gibbs’s sampling. We started with 200 and continuously increased the number of iterations to improve the consistency of the topics. We found 2000 to be the optimal number of iterations for this study dataset to maintain the accuracy of the data analysis and minimize the possibility of errors in the results.

**The number of keywords present.** This is the number of words most likely to be printed for each topic after model estimation (see Table 1). Due to the space limitation of the paper, here we show the top three keywords used in Twitter and Weibo posts. The co-occurring words are sorted from highest to lowest average fit.

**Topics clustering.** Through an analysis of the latent meaning of the keywords of each topic, we clustered all the topics into several topic homogeneous groups, namely issues (see the results section). This necessary cluster analysis step allowed us to evaluate the distribution of issues in the corpus and helped us to explain and infer the thematic evolution of patterns of discourse.

### 3.4. Temporal Analysis

In this part, we will focus on the temporal analysis of discourse and the aggregate corpus in three-day units to produce a regular time series to see how the meanings of issues may evolve over time [62]. Previous studies have examined online discourse from a time series perspective, revealing the evolution of issues over time [63,64]. Combined with the crisis lifecycle concept, the issues brought up in discourse may occur in the following scenarios: (a) a peak of issues that are rarely mentioned before or after, or (b) a period of continuous discussion without a peak, or (c) a fairly constant discussion with occasional spikes due to relevant events. Similar to the issue trending outlined above, although the method we proposed is not aimed at event prediction, we sought to verify whether the user comment time-based mode can reflect the events in a rise-and-fall temporal pattern.

### 3.5. Social Network Analysis

As a third methodology, this study adopts social network analysis (SNA) on social media networks to examine the interaction patterns of Twitter and Weibo accounts. As a common method for social media analysis, network analysis is used for a range of aspects, such as identifying bots, fake news, and opinion leaders [65,66]. The main purpose of this study is not to improve a new method or technique but to perform a comparison of the discourse communication patterns and opinion leaders between different social network environments. The interactions among these users are created when they reply to or mention one another. The network graph is comprised of nodes and lines, the nodes of a graph are considered to be actors, and the lines that link those actors are considered to show the correlation [67]. This exhibits the interactions between the various individuals (accounts) on social media, including mentioning, retweeting (reposting), and replying; through the graph, we can recognize the main groups and sub-groups of actors in a network during online discourse.

We conducted a social network analysis to examine the key influencers (opinion leaders) during the COVID-19 pandemic on Twitter and Weibo. We analyzed the relationships between key participants and the whole network of each influencer and identified the top-ranked influencers after the measurement. The social network analysis process followed three steps:

(1) Structure the relationship matrix of key influencers.
(2) Map the whole network of key influencers.
(3) Calculate the top-ranked influencers.

**(1) Structure the relationship matrix of key influencers.** Referring to several studies on social media actor relationship extraction [68,69], we selected all accounts containing “@” mentioning two or more accounts in the tweets/posts of the dataset as the sample data of key influencers. The total numbers of valid accounts after filtering was 206 Twitter accounts and 214 Weibo accounts. Then, we constructed a relationship matrix of the key influencer accounts on the Twitter and Weibo platforms (see Table 2 and Table 3).

In Table 2 and Table 3, it can be seen that the horizontal and vertical account numbers are identical and correspond in sequence. The intersection of the two is represented by 0 or 1, where 0 means no intersection and 1 means intersection. Based on this, we can identify which accounts generate connections between them [70,71,72]. The matrix data were also imported into UCINET 6 social network analysis software for the next analysis.

**(2) Map the whole network.** Next, we used the UCINET 6 software to map the whole network of Twitter and Weibo key influencer accounts. We also calculated the types of actors present in both graphs using different color schemes, defining them as “media, politicians, companies, etc.”. Finally, the percentage of key influencer types for both platforms was also counted in a comparison chart (see the results section).

**(3) Calculate the top-ranked influencers.** Secondly, we used the network to calculate the key actors to identify opinion leaders in the network. The main indicator for finding opinion leaders is the centrality of the network [50]. In social network analysis, several indicators/measurements show how the key actors’ network and wield influence within a social structure. The centrality measure is one of the most popular methods for calculating the centrality score for each node [67]. The individual in one unit represents the number of connections that this account has, which means that if any individual has a high centrality score this account (he/she/organization) may be identified as the most popular opinion leader, with the most connections with other actors in the group of networks. On the contrary, if it receives a low score, this means that the actor is on the periphery [67].

We introduced DegreeCent and Betweenness as our research indicators to describe the actor’s centrality. DegreeCent (degree centrality) is defined as the number of ties incident upon a node, which can indicate the relative importance of a node in the network [73]. A given node x is calculated as a ratio between the number of nodes connected with node x and the total number of nodes in the network (decreased by one) [74]. Betweenness (Betweenness centrality) captures the number of shortest paths between other nodes when passing through a given node. Betweenness can be regarded as a complementary measure of power, because it tends to capture actors’ actual access to resources [75]. Actors with a high betweenness can mediate and capitalize upon flows of information or other resources between disconnected actors [76].

## 4. Results

### 4.1. LDA Analysis Results

**Figure 1:**
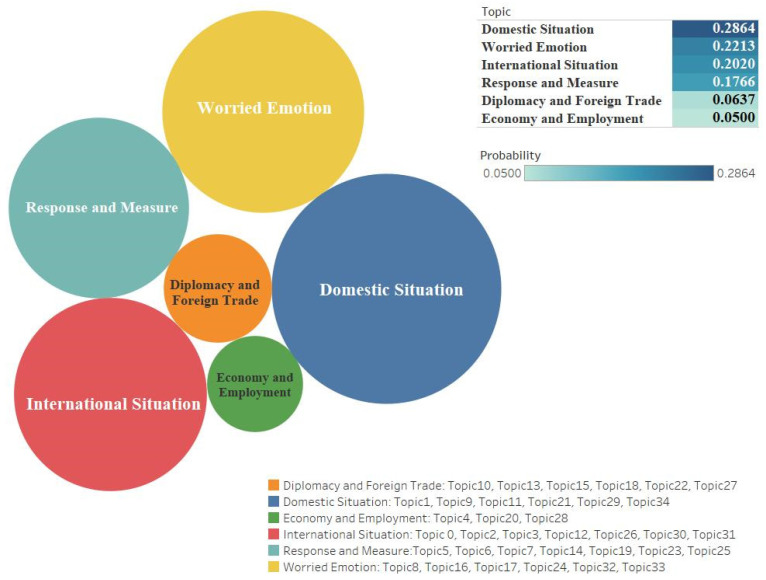
Clustering the topical issues, corresponding topics, and values of Twitter.

**Figure 2:**
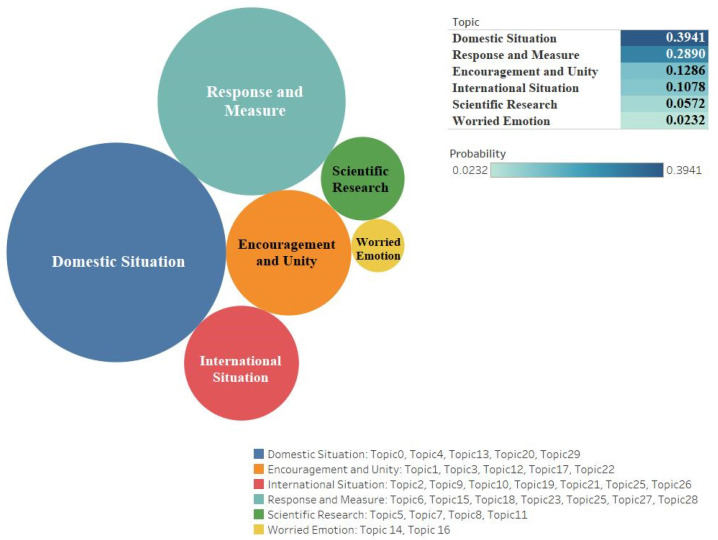
Clustering the topical issues, corresponding topics, and values of Weibo.

Through the semantic analysis of the keywords belonging to each topic, we identified six outstanding clustering topical issues from the dataset of two platforms. Figure 1 and Figure 2 show the results for issues, probability values, and corresponding topics.

From this analysis, we found that the two platform users gave similar attention to the issue ‘*Domestic situation*’. Twitter (n = 0.286391674) had the main keywords “virus outbreak, virus escalation, country, confirm, citizen, parade” and Weibo (n = 0.394134095) had the related keywords “epidemic, new case, cases of cure, confirmed, diagnosed, resumed production, discharge from hospital”. Both of those ranking first mainly reflect information updates and descriptive comments on the internal spread of the epidemic in each country. Besides the domestic situation, users also focused on issues related to the issue ‘*International situation*’ during the entire studied period. Twitter (n = 0.201997953) had keywords such as “WHO, offshore, global, cumulative, countries”. Weibo’s (n = 0.107792817) keywords included “Iran, Italy, increase, World Health Organization, foreign, million cases, Korea, outbreak, global”.

Secondly, the issue ‘*Response and measure*’ focused on implementing solutions and countermeasure suggestions; it gained some user attention. The probability values show that the cluster topic ranked four on Twitter (n = 0.176605722), as well as second on Weibo (n = 0.28899222). Keywords for both platforms include “vaccine, united, prepare, quarantine, released, control, source, attendance”, etc.

Thirdly, during the COVID-19 epidemic, it is not surprising that the issue ‘*Worried emotion*’ was the main concern on Twitter (n = 0.221291308). The keywords of this issue include “pessimistic status” and specific keywords such as “trigger, worry, doubt, anxiety, fear, lie” had a high probability of occurrence. In contrast, Weibo (n = 0.02323773) users focused on keywords such as “economic and social, induced, worry, doubt, anxiety, critical illness”, etc. This issue on Twitter is very high, but has the lowest proportion on Weibo. In contrast with this, users of the Weibo platform like to discuss the issue ‘*Encouragement and unity*’, which replaces the worry emotion to some extent; this is also a unique issue of the Weibo platform, emphasizing the promotion of positive and typical deeds.

Last but not least, we also verified other sets of issues corresponding to the platforms. Among them, Weibo includes the prominent issue, ‘*Scientific research*’, which is emphasized by using scientific research, impartial attitude for country’s response to the epidemic, and complete control of the outbreak through vaccine development and scientific experimental methods (related keywords include “research, expert, institution, Nanshan Zhong, University, experiments”). Regarding Twitter users’ special two issues, we found the issue ‘*Diplomacy and foreign trade*’ (n = 0.06371778); its representation keywords include “Trump, President, Democrats, Mike Pence, voters, Russia, prices”). There was also the issue ‘*Economy and employment*’ (n = 0.049995564); its corresponding keywords include “workers, seniors, money, industry, gas, oil”, expressing Twitter users’ concerns about the country’s foreign affairs, trade policy, social employment, and economic status. These specific issues also show the different value orientations of the two countries; for example, Twitter users are more concerned about the economy’s health, while Weibo users focus more on public health [77].

### 4.2. Temporal Analysis Results

Similar to tracking peaks in messages over time, we also examined the trends of issues over time. We compared the topics discussed on Twitter and Weibo in the context of the COVID-19 pandemic during the same period. By doing this, we identified the co-variation between a topical trend in the social media networks and changes in the real world, as well as the differences between the two platforms. Finally, after aggregating the temporal trends of each issue, we constructed a temporal heat map of all the issues.

**Figure 3:**
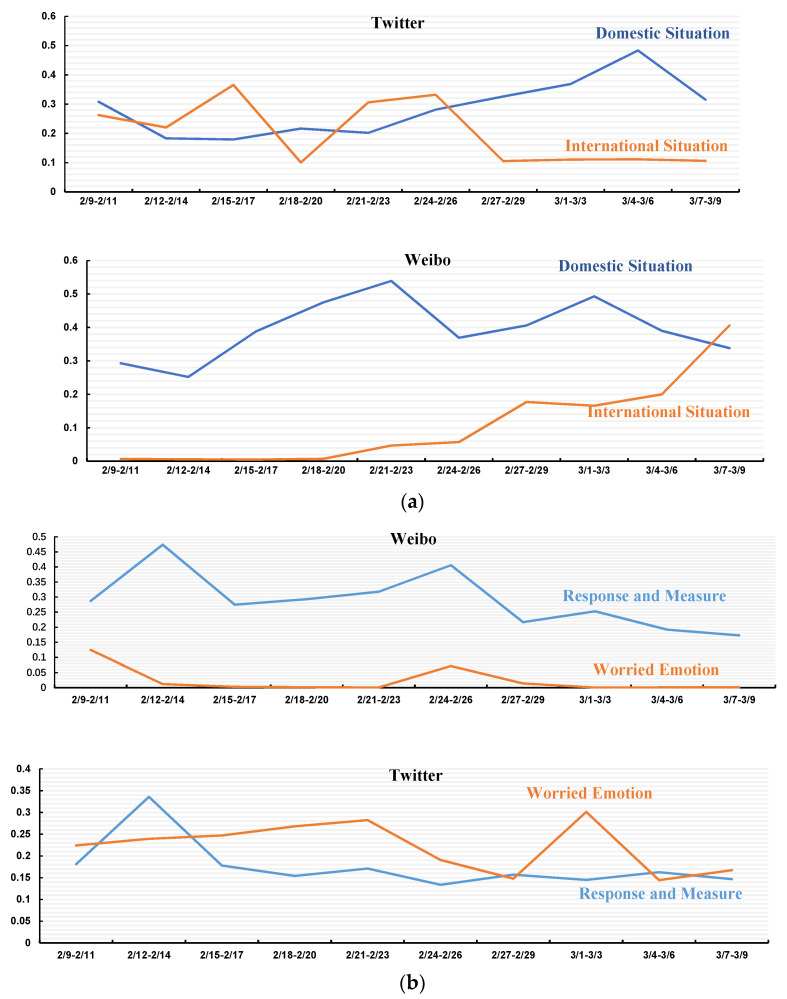
Issues (**a**): Domestic situation/international situation on Twitter and Weibo; Issues (**b**): Response and measure/worried emotion on Twitter and Weibo.

Figure 3a,b shows the topic time trend graphs for Weibo and Twitter, respectively. Here. the horizontal axis represents the unit period of three days, with the total time interval being the same as that used in the topical analysis (9 February to 9 March 2020), while the vertical axis represents the value of the issue’s probability of occurrence at specific moments in time. This value can be between 0 and 0.6. To facilitate the comparison, we mainly compared the same classification of issues from the two platforms (a) and (b) in turn.

Figure 3a depicts the trends on Weibo/Twitter over time during the outbreak of COVID-19, which started to spread in China in early 2020, until March. Although the international outbreak also began to emerge and spread on a small scale during this month, Weibo users were much more concerned about the Chinese outbreak than an international pandemic. The situation started to change from “2.21–2.23”. As the epidemic in China gradually declined but the situation became worse in Italy, South Korea, and the United States, Weibo began to see more discussion about the international pandemic in early March (3.7–3.9).

In contrast, Twitter users’ early focus on their home country (the US) remained largely consistent with the foreign outbreak, but in contrast to the Weibo platform the level of discussion changed significantly as the domestic situation evolved (between 2.24 and 2.26); the attention given to the domestic situation continued to rise, while discussion of the international situation continued to fall. As such, this result can also confirm the rationality of using temporal analysis for social media topic tracking.

In Figure 3b, it can be seen that, except for a small peak in *Worried emotion* during the “2.21–2.26” period, Weibo users maintain a constant downward trend, rarely posting negative comments about the epidemic. Instead, there are many positive comments concerning *Response and measure*. Twitter users show a significant difference from Weibo users concerning these two issues, with *Worried emotion* showing a continuous increase from “2.9–2.11” to “2.21–2.23”. Perhaps reflecting the fact that many people felt depressed at this time, this orange line reached a peak from “3.1–3.3”. By contrast, *Response and measure* remained flat from “2.15–2.17” to “3.7–3.9”. Although direct comparison between online opinion and the real world is not easy, given the different lifecycles of COVID-19 in China and the US, the way the probability distribution in the dynamically changing dataset increases/decreases as the quantity of related issues increases/decreases can be used as a valid indicator to track the reality in real time.

### 4.3. Social Network Analysis Results

**Figure 4:**
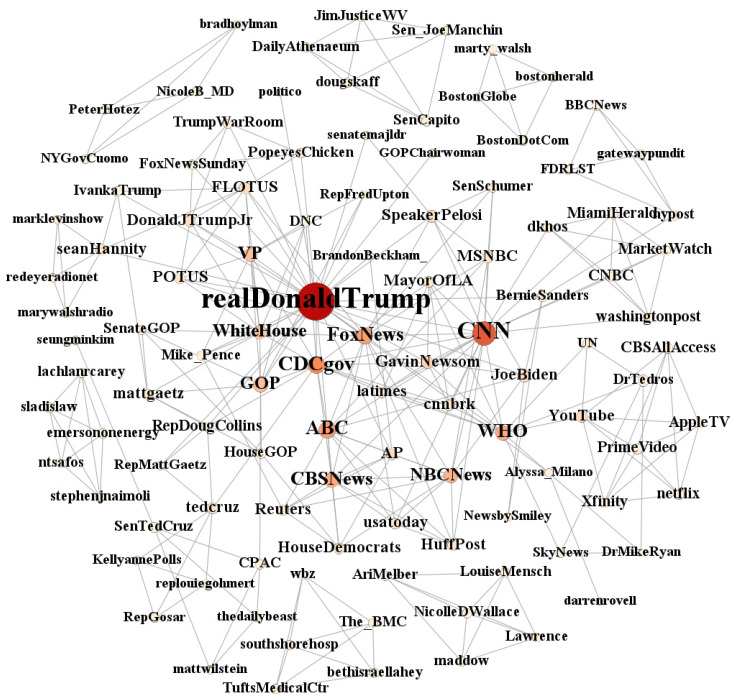
Whole network of Twitter.

After the SNA, Figure 4 shows the network of key actors related to COVID-19 on Twitter. The most connected node is the former president Donald Trump’s personal Twitter account (@realDonalTrump), which served as the largest central point of opinion leaders, controlling and influencing the topics of accounts from the surrounding circles. This account is closely surrounded by three types of opinion leaders: The first is the official accounts of media corporations in the United States, including @CNN, @ABC, @ABC News, @CBS News, and @FoxNews. The second is the personal Twitter accounts of key staff members of the U.S. government leaders, such as @Mike_Pence, the personal account of the former U.S. Former Vice President Mike Pence; current president Joe Biden; Speaker of the House @SpeakerPelosi; Senator @SenTedcruz; and @BernieSanders.

The third is the official accounts of relevant U.S. government departments, such as the White House @WhiteHouse, the Republican Party @GOP, the U.S. Department of Health and Human Services @CDCgov, and the Democratic Party @House Democracy. Finally, the network also includes an intertwined coalition of various other types of accounts, including the official World Health Organization @WHO and individual corporate accounts such as @YouTube and @Apple TV.

**Figure 5:**
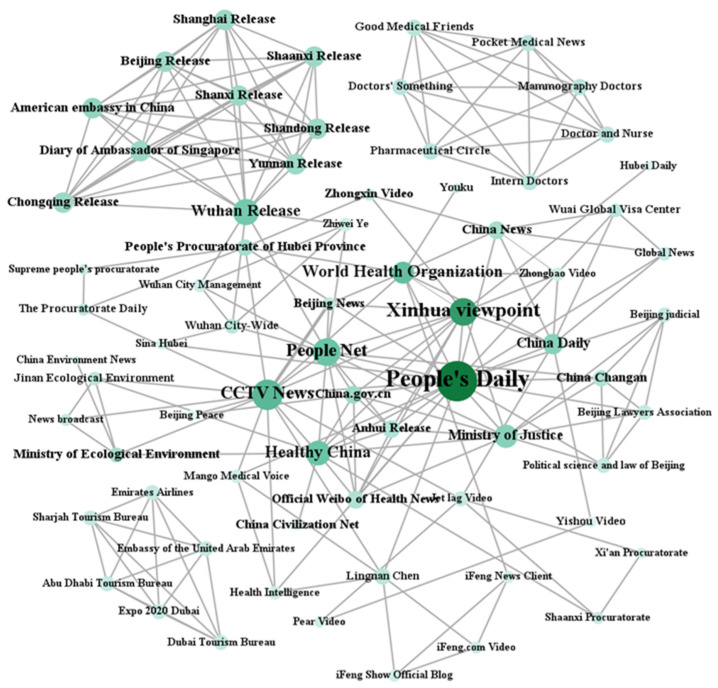
Whole network of Weibo.

The results of Figure 5 show that the opinion leaders in Weibo are mainly concentrated in three major sections: first, the core central layer (e.g., @People’s Daily, @CCTV News, @Xinhua Viewpoint, @World Health Organization, @HealthChina, etc.), with China’s official media as the core and co-existing with the official account of the WHO; second, “XX Release”, which is used by local governments to issue updates on the latest news, such as @Wuhan release, @Shanghai release, @Beijing release, @Shandong release, etc.; third, the professional consultation layer, which includes hospitals, doctors, and medical health professionals as the core actors (such as @Mammography Doctor, @Doctor’s Something, @Pharmaceutical Circle, @Palmed Medical News. These three sub-circles are at the center of Weibo’s opinion leader accounts and have the highest number of connections. At the same time, the network periphery is also surrounded by some relatively minor opinion leader alliances, including a local government accounts circle shown in the top left of Figure 5, a procuratorate alliance circle on the right, a medical sector-related alliance circle on the top right, and an alliance circle formed by Weibo accounts opened in China by the United Arab Emirates at the bottom left of the figure. This shows the scope of influence of COVID-19 and what these related departments respond to.

According to the results of the UCINET calculation of the centrality of networks (Table 4), on Twitter, US President Donald Trump, CNN News, and the World Health Organization become the most influential opinion leader accounts. Trump (@realDonaldTrump) was the first influencer and was more than twice as influential as the next nodes in during the COVID-19 pandemic, followed by CNN News (@CNN), which is a private news media organization. Additionally, in third place was the official account of the World Health Organization (@WHO). There is a significant relationship between the level of activity of the country’s current political activists, with a total of five of the top 10 being politicians such as the current US President Joe Biden (@JoeBiden), Congressman Ted Cruz (@SenTedcruz), US House Speaker Pelosi (@speakerPelosei); two being political party accounts for the Department of Health and Human Services (@CDCgov) and the Republican Party of the United States (@GOP); and two being news media accounts, @CNN and @ABC. This shows that, on the Twitter platform, political actors play a mainstream role in impacting public opinion. Corresponding to this in the Weibo network alliance, we found that China’s official media organizations @People’s Daily and @Xinhua Viewpoint were the most influential in terms of the overall structure of the network, ranking in the top two, while the third and fourth were @CCTV News and @World Health Organization, respectively. According to the statistics of the average ranking of the top 10 opinion leaders in terms of influence, six were official media and three were official accounts of the central government. In the China context, the official media accounts were conceived as the official “mouthpiece” or “transmission belt” of the official party ideology and government policies [78]. Other major institutions ranking in the top 10 in terms of influence included @People’s Daily, @HealthChina, @WuhanPublished, @ChinaDaily, @HubeiPeople’s Procuratorate, and @ChinaCivilization.com.

## 5. Conclusions

By applying text analysis, temporal analysis, and social network analysis, this comparative study probed how the public expresses opinions through social media; we detected the information flow during the COVID-19 pandemic within different platforms from China and the US. Through researching the online content and active actors, this study demonstrates several significant findings. Firstly, through LDA analysis, we found that, in the predominantly US social networks, represented by Twitter, participants were more concerned about the impact of COVID-19 on the economy and science and technology issues. Meanwhile, in the other social media sphere, represented by Weibo in China, politics and public health development were always the center of the discussion. A possible explanation for this is that the two governments applied different measures to control the spread of the virus during the early stages of the crisis. The US government used a soft and moderate approach at first—namely, a herd immunity strategy—in order to avoid seriously affecting economic development and people’s personal lives or work. China, on the other hand, applied more forceful measures, such as lockdowns, trajectory tracking, and strict quarantines. Thus, public health was protected at the cost of the decline of the economy and changes in people’s daily lifestyles [10]. This finding is relatively different to that of some other existing research based on the analysis of single social media platforms such as Twitter or Weibo [31,32,34,35,36]. This could be due to the fact that the data from these studies were drawn from different periods of the COVID-19 pandemic, meaning that their focus varies. Furthermore, it is important to note that, from a comparison perspective, the significant secondary concerns of the two sides are more likely to be prominent than a single perspective.

Secondly, we found that combining the results of LDA and temporal analysis, such as for the issues of *Domestic Situation* and *International Situation*, which appear alternately on Twitter and Weibo, to some extent reveals that online discourse reflects the crisis lifecycle according to the different stage of COVID-19 occurring in China and the US. However, the differences in the level of discussion of positive issues such as *Response and Measure* and negative issues such as *Worried Emotion* in different countries reflect their differences in society, value, cultural background, health communication, and administration systems in the real world beyond the social media ‘sphere’. For example, when facing the crisis, the Chinese government tended to launch campaigns through social media to inform and motivate the public [22]. The significance of the public comment framework in different social media contexts has been vividly debated within research on online media [79,80], which echoes the results of previous literature, such as how Weibo users tend to be more positive than Twitter users and the Chinese tendency for collectivism rather than individualism [24].

Thirdly, through the SNA technique, we were able to better discover the opinion leaders in different political backgrounds and conclude the pattern of the information communication actors’ network. This enabled us to better understand the dynamics of the discourse and to identify the opinion leaders without any preconceived views. The difference was that, in the US, political leaders played more important roles than the media. They tended to directly express their opinion and emotion. Since the United States is a multi-party system and different opinion leaders have different views and numbers of followers, the clusters of the discourse network were polycentric. However, in China the official media and government accounts mainly controlled the release of information, meaning that the main structures of opinion leaders’ networks were more concentrated. Additionally, the most important opinion leaders followed a hierarchical order, with central media occupying a more essential position than the local media, which is determined by China’s top-down and unitary administrative system [5]. This also strengthens the result of Lin et al. [22] and Gao et al. [23], who found that the US praises individualism while China upholds collectivism.

Taken together, the findings discussed here offer a glimpse into the content and structure of discourse from social media platforms, such as Twitter and Weibo, which may vary in terms of crisis development and different cultures in the context of COVID-19. This study contributes to the small body of comparative research on this topic; we provide methods and concepts for an international comparative perspective to fill this gap and to expand our understanding of social media functions within different culture domains. This research also has some limitations. Firstly, because of the platform filter function and censorship, the data we acquired may be not incomplete, thus the research sample may be not comprehensive. Secondly, this study does not expand more on the comparison of arithmetic automation on opinion manipulation between the two representative countries. Thirdly, in further analysis, semantic network analysis should be combined with temporal analysis to explore the details of discourse trends.

## Figures and Tables

**Table 1:** Twitter and Weibo topics and keywords.

**(a) Twitter**				
**Topic 0**	**Topic 1**	**Topic 2**	**Topic 3**	**Topic 4**
Wuhan	covidoutbreak	UK	inter	workers
infected	US	emergency	Lombardia	elderly
patience	trump	Iran	lockdown	industry
…	…	…	…	…
**Topic 5**	**Topic 6**	**Topic 7**	**Topic 8**	**Topic 9**
vaccine	quarantine	epidemic	dangerous	city
united	released	prevention	violence	reported
prepare	test	discharged	gain	truth
…	…	…	…	…
**Topic 10**	**Topic 11**	**Topic 12**	**Topic 13**	**Topic 14**
response	American	Brazil	declares	community
California	Texas	pandemic	Pence	deploys
democrats	event	Tenerife	constituents	minister
…	…	…	…	…
**Topic 15**	**Topic 16**	**Topic 17**	**Topic 18**	**Topic 19**
Russia	race	hoax	president	fight
markets	lies	Donald	leader	school
CNN	trash	deaths	confirmed	close
…	…	…	…	…
**Topic 20**	**Topic 21**	**Topic 22**	**Topic 23**	**Topic 24**
destroy	airport	Asia	quarantined	fears
oil	populism	trade	fight	spread
funding	Obama	business	treat	contagious
…	…	…	…	…
**Topic 25**	**Topic 26**	**Topic 27**	**Topic 28**	**Topic 29**
source	Russia	Trump	gas	covidupdate
response	carnival	president	oil	confirms
wash-hands	covidoutbreak	announced	conservative	concern
…	…	…	…	…
**Topic 30**	**Topic 31**	**Topic 32**	**Topic 33**	**Topic 34**
pandemic	Africa	fears	deaths	Seattle
diamond	Egypt	outbreak	live	congress
ship	rages	symptoms	breaking	coronaalert
…	…	…	…	…
**(b) Weibo**				
**Topic 0**	**Topic 1**	**Topic 2**	**Topic 3**	**Topic 4**
epidemic	good news	Italy	martyr	epidemic-prevention
confirmed	united	World Health Organization	medical-staff	committee
cases	support	overseas	donation	Adjust
…	…	…	…	…
**Topic 5**	**Topic 6**	**Topic 7**	**Topic 8**	**Topic 9**
research	implementation	Nanshan Zhong	send for testing	Iran
expert	accurate	University	clinical Trials	Italy
institution	guarantee	promote	experiments	increase
…	…	…	…	…
**Topic 10**	**Topic 11**	**Topic 12**	**Topic 13**	**Topic 14**
World Health Organization foreign million cases …	autopsy human remains expert panel …	Wenhong Zhang Lei Feng Spirit advanced-individual …	designated-hospitals infected-persons staff …	economic and social induced worry …
**Topic 15**	**Topic 16**	**Topic 17**	**Topic 18**	**Topic 19**
level 1	doubt	donation	control	Korea
prevention	anxiety	plasma	source	outbreak
response	critical illness	medical staff	attendance	global
…	…	…	…	…
**Topic 20**	**Topic 21**	**Topic 22**	**Topic 23**	**Topic 24**
work resumption	global	offer	treatment	medical team
labor resumption	organization	donation	discharge	prevention
leave hospital	import	blood plasma	declaration	command
…	…	…	…	…
**Topic 25**	**Topic 26**	**Topic 27**	**Topic 28**	**Topic 29**
France	death	services	isolation	outbreak
global	region	leadership	treatment	cure
report	overseas	National Health Commission	detection	resume work
…	…	…	…	…

**Table 2:** Relationship matrix of key influencer accounts (206 for Twitter and 183 for Weibo).

	01 @realDonaldTrump	02 @GOP	03 @FoxNews	04 @WHO	05 @CDCgov	…
01 @realDonaldTrump	0	1	1	1	1	…
02 @GOP	1	0	0	0	1	…
03 @FoxNews	1	0	0	0	0	…
04 @WHO	1	0	0	0	0	…
05 @CDCgov	1	1	0	0	0	…
06 @ABC	1	0	1	0	1	…
07 @CNN	1	0	1	1	1	…
…	…	…	…	…	…	…
206 @seanHannity	1	0	0	0	0	…

**Table 3:** Relationship matrix of the 183 Weibo key influencer accounts.

	01 @People’s daily	02 @CCTV News	03 @WHO	04 @gov.cn	…
01 @People’s daily	0	1	0	1	…
02 @CCTV News	1	0	1	1	…
03 @WHO	0	1	0	0	…
04 @gov.cn	1	1	0	0	…
05 @Wuhan Release	0	1	0	1	…
06 @Shimian	0	1	0	0	…
07 @Xinhua viewpoint					
…	…	…	…	…	…
183 @Healthy China	1	0	0	1	…

**Table 4:** Network centrality measurements on Twitter and Weibo.

Weibo				Twitter			
User’s Name	*DegreeCent*	*Betweenness*	Rank	User’s Name	*DegreeCent*	*Betweenness*	Rank
@People’s Daily	28.000	6021.398	1	@realDonaldTrump	58.000	8316.265	1
@Xinhua Viewpoint	24.000	6730.424	2	@CNN	26.000	1845.803	2
@CCTV News	19.000	5822.823	3	@WHO	14.000	2280.219	3
@WHO	17.000	4390.561	4	@CDCgov	16.000	1137.272	4
@people.com.cn	16.000	5278.575	5	@GOP	12.000	1442.524	5
@Health China	15.000	3916.136	6	@JoeBiden	12.000	784.282	6
@Wuhan Release	14.000	3358.000	7	@ABC	15.000	601.810	7
@China Daily	11.000	1896.098	8	@SenTedcruz	7.000	742.000	8
@Hubei People’s	9.000	1240.625	9	@SpeakerPelosi	8.000	226.117	9
@ChinaCivilization.com	8.000	1478.000	10	@CPAC	4.000	1152.000	10
……	/	/	/	……	/	/	/
Minimum	1.000	2.000	/	Minimum	1.000	0.500	/
Maximum	28.000	6730.424	/	Maximum	58.000	8316.265	/

## Data Availability

The data presented in this study are available on request from the corresponding author.
